# The effects of torsion on horizontal motor fusion and stereopsis

**DOI:** 10.1038/s41598-023-28169-z

**Published:** 2023-01-16

**Authors:** Tongzhu Feng, Yueping Li, Wei Zhang

**Affiliations:** 1 General Hospital of Northern Theatre Command, Shenyang, 110000 China; 2 Tianjin Eye Hospital, Tianjin Key Laboratory of Ophthalmology and Vision Science, Clinical College of Ophthalmology of Tianjin Medical University, Tianjin, 300020 China

**Keywords:** Medical research, Risk factors, Signs and symptoms

## Abstract

To investigate the effects of ocular torsion on horizontal motor fusion and stereopsis in normal adults and to probe the effects of torsion on peripheral fusion, macular fusion and foveal fusion. Twenty-five normal adults aged 30–38 were enrolled in this study. During the synoptophore assessment, the break points (BP) and recovery points (RP) of convergent fusion (CF) and divergent fusion (DF) and random-dots stereopsis were measured and analyzed at intorsion and extorsion of 3°, 5°, 7°, and 9°. According to the different sizes of the retinal areas stimulated by the synoptophore slides, fusion was classified into three categories: peripheral fusion (p-F), macular fusion (m-F) and foveal fusion (f-F). The p-F, m-F and f-F were analyzed and compared at the same torsional angle. There were significant differences in BPCF, RPCF, BPDF and RPDF among different torsion angles (ANOVA, P < 0.05). The Tukey's multiple comparison test showed that BPCF and RPCF of p-F, m-F and f-F decreased significantly at extorsion and intorsion ≥ 5°, compared with baseline (0° torsion) (P < 0.05). Compared with the baseline, BP of DF decreased significantly at torsion angles ≥ 3°, ≥ 5° and ≥ 7° for p-F, m-F, and f-F, respectively (P < 0.05), and RP of DF decreased significantly at torsion angles ≥ 5°, ≥ 9° and ≥ 7° for p-F, m-F, and f-F, respectively (P < 0.05). Comparison among p-F, m-F and f-F revealed significant differences only in BPCF at an intorsion of 3°, extorsion of 3° and the baseline (ANOVA, P < 0.05). There was a significant difference in the proportion of subjects with different sizes of RDS at different torsional angles (Fisher's exact test, P = 0.000). Fine stereopsis was damaged with increasing torsion. Torsion within the normal range of cyclofusion affects the horizontal motor fusion of convergent and divergent fusion and stereopsis. Torsion ≥ 5° should be considered during strabismus surgery for regaining fine binocular vision.

## Introduction

It is well-established that rotation is mainly influenced by the central system, the otovestibular system, and the retinal stabilization system^1^. Torsional diplopia has been documented in 94% of patients with brainstem lesions^2^. Superior oblique palsy is the most common disorder in cyclodeviation^3,4^. Healthy adults have a wide amplitude of cyclofusion with a mean excyclofusion of 16.8° and a mean incyclofusion of 11.8°^5,6^. An increasing body of evidence suggests that torsional diplopia can be overcome with a cyclofusion of 6 to 10°^6,7^. Besides, cyclofusion can cause dizziness, blurred vision and diplopia. It is widely thought that ocular torsion could affect horizontal fusion and stereovision^8^. Guyton et al. suggested that cyclofusion had two components: motor and sensory fusion^9^. It is well-established that cyclofusion in the peripheral visual field is larger than in the central visual field^10,11^. Cyclotorsional fusion is also crucial for maintaining binocular vision. Few previous studies investigated the effect of torsion with the different directions and angles on binocular vision in the peripheral and central visual fields. In the present study, we used a synoptophore to simulate objective torsion within the normal amplitude of cyclofusion and probe the changes in horizontal motor fusion in different visual fields and stereopsis in healthy adults.

## Subjects and methods

### Subjects

This study conformed to the ethical principles of the Declaration of Helsinki for medical research involving human subjects. The protocol and waiver of informed consent used in this study were approved by the Institutional Review Board (IRB) of Tianjin Eye Hospital (TJEH). Twenty-five normal adults, 9 males and 16 females, aged 30–38 years (mean 34.4 years), were enrolled. All subjects underwent comprehensive examinations, including refractive error, corrected visual acuity, slit lamp, fundus examination, deviation, binocular fusion, ocular motility and stereopsis.

The inclusion criteria were: (1) patients without amblyopia, anisometropia, manifest strabismus, motility disturbance, nystagmus and no systemic neurological disease; (2) measured by alternative prism cover test, deviation ranged from 4 prism dioptor (PD) esophoria to 5PD exophoria; (3) tested by prism bar, the amplitudes of convergent fusion (CF), divergent fusion (DF) and vertical fusion were 25 to 30PD, 6 to 8PD and 2 to 3pd, respectively; (4) cyclofusion ranged 15–25° tested by synoptophore^3^; (4) stereo acuity of 40 to 50 s of arc by Titmus testing.

### Examinations

**Figure 1:**
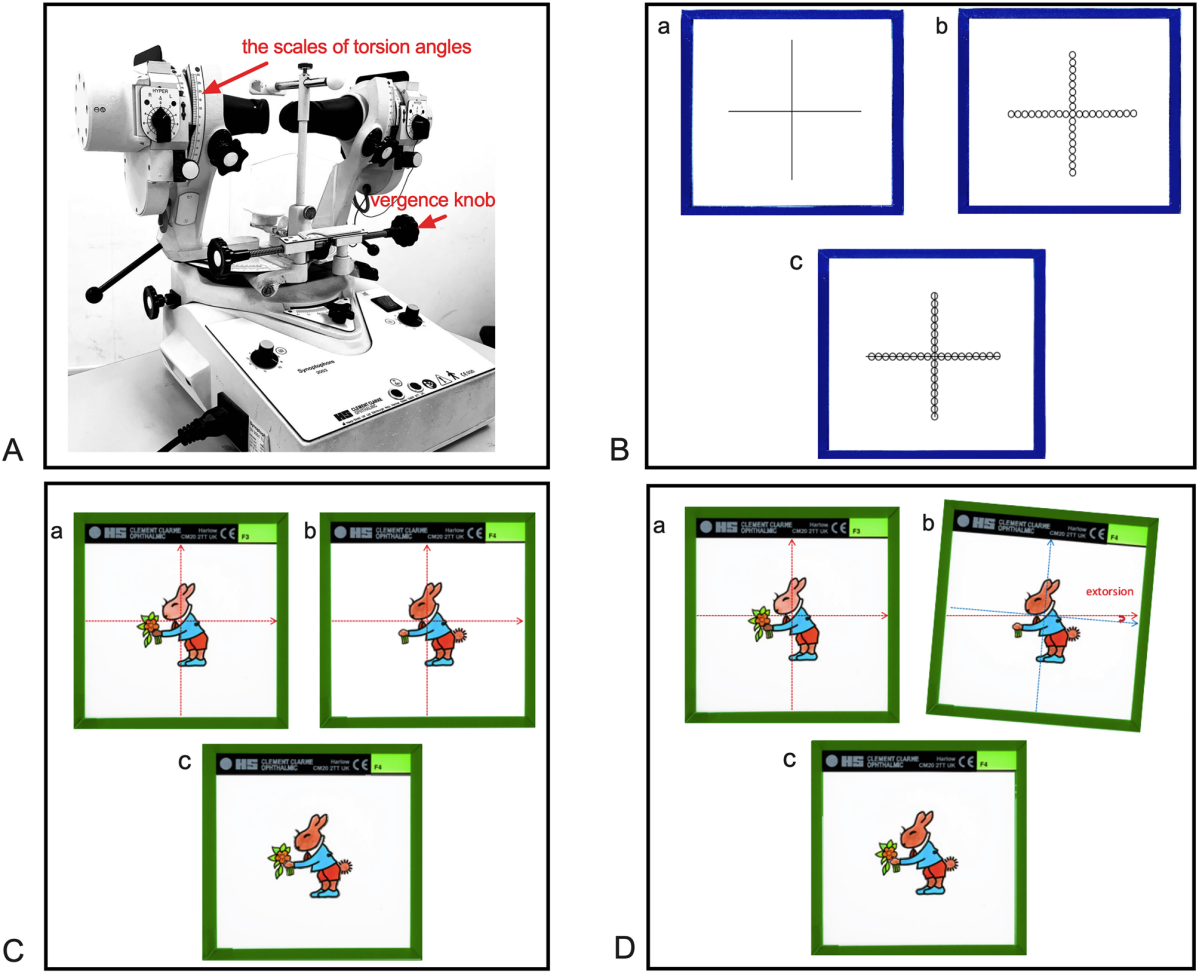
The steps of assessing fusion amplitude by synoptophore. (**A**) The scales of torsional angles and vergence knob showing in the picture of synoptophore. (**B**) The original objective deviation and torsion were determined by a pair of synoptophore "cross" slides. (a) the image presenting to the righ eye; (b) the image presenting to the left eye; (c) The subject pushes the rob till to gain the image of simultaneous perception. (**C**) A pair of fusion slides (11° slides) and fusion. (a) the image with one "control" presenting to the righ eye; (b) the similiar image with the other "control" presenting to the left eye; (c) The subject fuses and gains the single and complete image with two "controls" in the position of the original objective deviation. (**D**) A pair of fusion slides at a simulated torsional angle and fusion. (a) the image presenting to the righ eye; (b) the similiar image at a simulated extorsion angle presenting to the left eye; (c) the subject fuses and gains the single and complete image.

All examinations were performed by the same ophthalmologist. The methods and steps for assessing the amplitude of horizontal motor fusion by the synoptophore (Fig. 1A) were as follows:

1. The original objective deviation and torsion were determined by the synoptophore "cross" slides (Fig. 1B). Then the arms were set at the subject's angle of deviation to measure fusional amplitude from the phoria position.
2. After presenting the synoptophore fusion slides (a pair of similar images with an incomplete "control"), a single and complete image was obtained at position (1) (Fig. 1C). The examiner turned the fusion knob slowly from position (1) and recorded break points (BP) until the subject reported diplopia, at which point the two images were suddenly far apart or reported one control suddenly disappeared. We used three different sizes of fusion slides, 1° for foveal fusion (f-F), 5° for macular fusion (m-F) and 11° for peripheral fusion (p-F).
3. Then the examiner slowly turned the knob inversely and recorded recovery points (RP) until a single and complete image was regained. During the measurement process, the examiner observed the subject's vergence movement to guarantee the reliability of the subjective description.
4. All BPs and RPs of CF and DF at intorsion and extorsion 0°(set as the baseline), 3°, 5°, 7°, and 9° were measured three times (Fig. 1D). The average values were recorded.

**Figure 2:**
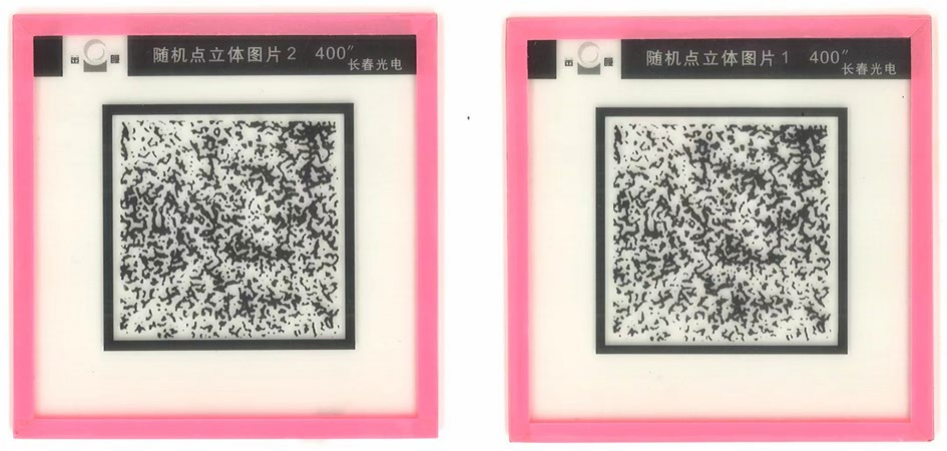
A pair of synoptophore slides with random-dots stereopsis.

The methods and steps of measurement of stereopsis using the synoptophore slides of random-dots stereopsis were as follows:

1. The original objective deviation and torsion were determined by using the synoptophore "cross" slide (Fig. 1B). Then the arms were set at the subject's angle of deviation
2. Then the slides with random-dot stereopsis (RDS) were set to position (1) (Fig. 2); the subjects identified the figure embedded in RDS slides at intorsion and extorsion of 0°(as baseline), 3°, 5°, 7°, and 9°. The grades of parallax in RDS for synoptophore testing in this study were: 400″, 80″, and 30″. We recorded “nil” as no stereovision.

### Statistical analysis

Statistical analyses were carried out by SPSS Statistics 22.0 (IBM, Armonk, United States). One-way ANOVA was applied to compare BPCF, RPCF, BPDF and RPDF of the fusion slides with different torsional angles and at the same simulated torsion among peripheral fusion, macular fusion and foveal fusion. Then Tukey’s multiple comparison tests were applied. Fisher’s exact test was used to analyze the proportion of patients with different sizes of RDS at different torsion angles. A p-value < 0.05 was statistically significant.

## Results

### Horizontal fusion and stereopsis at baseline (no torsion)

A total of 25 adults were included in the present study, and the average spherical equivalent of refractive error was − 2.25 ± 0.25D (− 0.50D to − 6.00D). The BP and RP of incyclofusion ranged from 12.0° to 21.5° (mean 16.35° ± 3.10°) and from 8.0° to 13.0° (mean 10.05° ± 2.20°). The BP and RP of excyclofusion ranged from 11.5° to 23.0°(mean 15.85° ± 2.80°) and from 8.0° to 12° (mean 10.1° ± 2.10°). All subjects had an RDS of 30″ tested by synoptophore slides.

### The effect of torsions on horizontal fusion

#### Peripheral fusion

**Figure 3:**
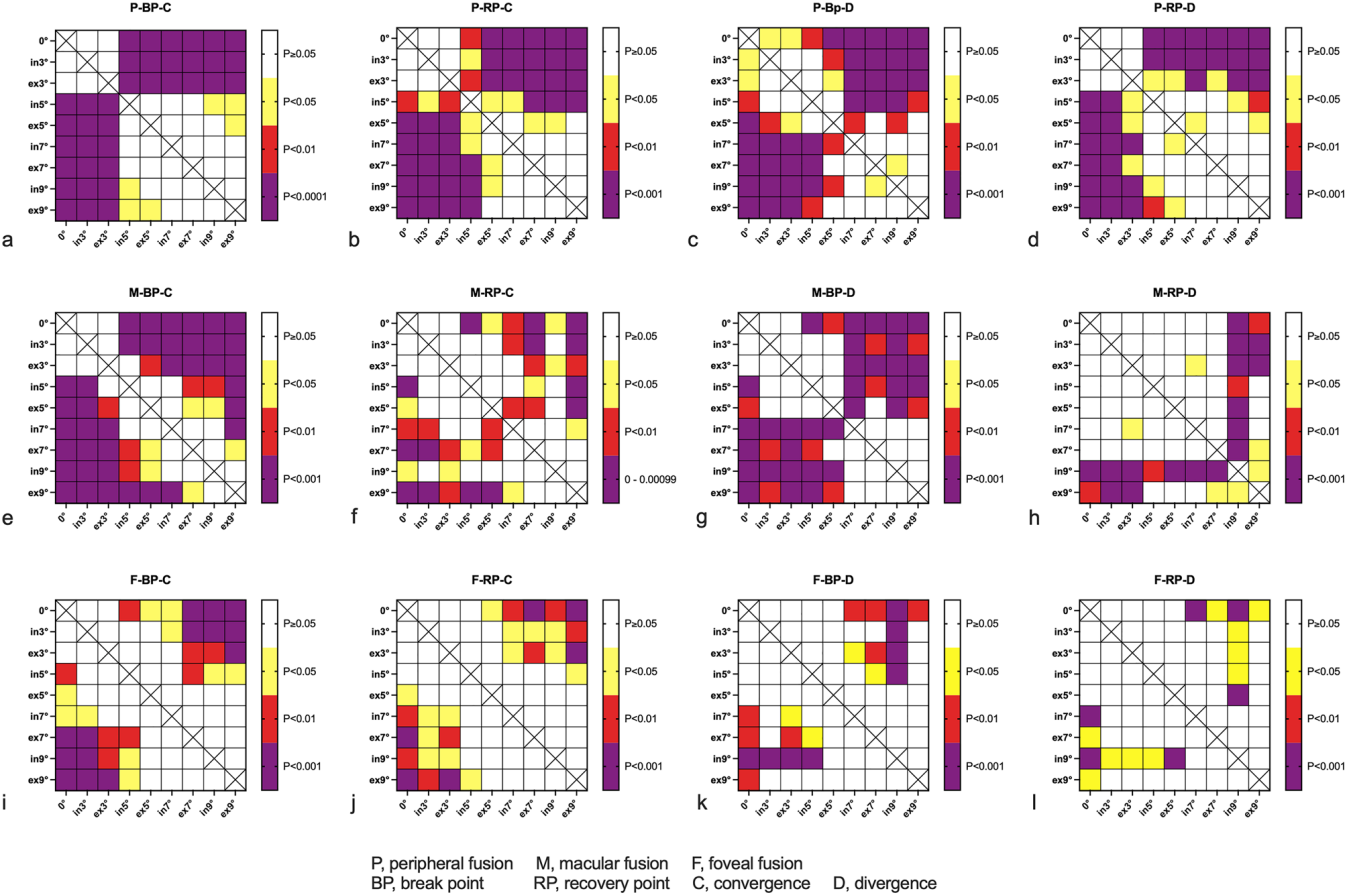
The results of the Tukey multiple comparison tests. (**a**) the results of break points of convergent fusion of peripheral field; (**b**) the results of recovery points of convergent fusion of peripheral field; (**c**) the results of break points of divergent fusion of peripheral field; (**d**) the results of recovery points of divergent fusion of peripheral field; (**e**) the results of break points of convergent fusion of macular field; (**f**) the results of recovery points of convergent fusion of macular field; (**g**) the results of break points of divergent fusion of macular field; (**h**) the results of recovery points of divergent fusion of macular field; (**i**) the results of break points of convergent fusion of foveal field; (**j**) the results of recovery points of convergent fusion of foveal field; (**k**) the results of break points of divergent fusion of foveal field; (**l**) the results of recovery points of divergent fusion of foveal field.

The average values and ranges of BPCF, RPCF, BPDF and RPDF are shown in Table 1. There were significant differences in BPCF, RPCF, BPDF and RPDF at different torsion angles (ANOVA test, F = 21.979, 10.200, 12.303, 11.308; P = 0.000, 0.000, 0.000, 0.000, respectively). Furthermore, the Tukey multiple comparison test showed that the average values of BPCF, RPCF and RPDF at extorsion and intorsion angles of 5°, 7°, and 9° decreased significantly compared to the baseline (0°torsion) (P < 0.05) (Fig. 3a–d). The BPDF decreased significantly at all simulated torsions (P < 0.05). The fusional vergence amplitudes of peripheral fusion gradually reduced as the degree of torsion increased.

**Table 1:** BPCF, RPCF, BPDF and RPDF of p-F at different rotations.

Torsion	Convergent fusion (CF) n = 25		Divergent fusion (DF) n = 25	
	BPCF (°)	RPCF (°)	BPDF (°)	RPDF (°)
ex9°	9.22 ± 5.38^a^	4.80 ± 3.89^a^	2.80 ± 1. 45^a^	0.75 ± 0.97^a^
	[1.0, 18.5 ]	[0.0, 12.0]	[0.0, 6.0]	[0.0, 3.0]
ex7°	10.58 ± 3.43^a^	5.85 ± 3.27^a^	3.08 ± 1.24^a^	1.20 ± 0.77^a^
	[5.0, 19.5]	[2.0, 15.5]	[1.0, 5.0]	[0.0, 2.0]
ex5°	13.35 ± 5.42^a^	6.88 ± 3.21^a^	3.70 ± 1.98^a^	1.30 ± 1.08^a^
	[6.5, 22.0]	[2.0, 17.5]	[1.5, 8.0]	[0.0, 3.0]
ex3°	19.90 ± 3.84	11.25 ± 4.57	4.85 ± 1.22^a^	2.25 ± 1.36
	[12.5, 28.0]	[7.0, 21.0]	[3.0, 8.0]	[1.0, 4.0]
0°	20.35 ± 4.40	11.50 ± 4.69	5.58 ± 1.42	2.45 ± 0.18
	[12.0, 28.0]	[7.0, 20.0]	[3.0, 8.5]	[1.5, 4.0]
in3°	20.07 ± 4.27	11.37 ± 4.71	5.05 ± 1.31^a^	2.45 ± 1.18
	[11.0, 28.5]	[6.0, 19.5]	[3.0, 8.0]	[1.0, 3.5]
in5°	13.85 ± 4.10^a^	8.50 ± 4.11^a^	4.70 ± 1.89^a^	1.45 ± 1.05^a^
	[7.0, 22.0]	[4.0, 18.0]	[2.0, 7.5]	[0.0, 3.0]
in7°	10.93 ± 3.93^a^	6.50 ± 3.12^a^	2.80 ± 1.40^a^	0.85 ± 0.79^a^
	[5.5, 18.0 ]	[2.0, 14.0]	[1.0, 6.5]	[0.0, 2.0]
in9°	9.35 ± 4.15^a^	5.17 ± 3.71^a^	2.30 ± 1.13^a^	0.75 ± 0.97^a^
	[4.0, 20.0]	[1.0, 12.0]	[0.0, 4.0]	[0.0, 3.0]
F	21.979	10.200	12.303	11.308
P	0.000	0.000	0.000	0.000

ex, extorsion; in, intorsion.

^a^P < 0.05 comparison with the value in the baseline (0°) by Tukey multiple comparison tests.

#### Macular fusion

The average values and ranges of BPCF, RPCF, BPDF and RPDF are shown in Table 2. There were significant differences in BPCF, RPCF, BPDF and RPDF at different torsional angles (ANOVA test, F = 8.965, 13.929, 5.737, 3.358; P = 0.000, 0.000, 0.000, 0.001, respectively). Besides, the Tukey multiple comparison test showed that the average values of BPCF, RPCF and BPDF at extorsion and intorsion angles of 5°, 7°, and 9° decreased significantly compared to baseline (P < 0.05) (Fig. 3e–k). The average RPDF values at extorsion of 9° and intorsion of 9° decreased significantly compared with baseline (P < 0.05). The fusional vergence amplitudes of macular fusion gradually decreased as the degree of torsion increased.

**Table 2:** BPCF, RPCF, BPDF and RPDF of m-F at different rotations.

Torsion	Convergent fusion (CF) n = 25		Divergent fusion (DF) n = 25	
	BPCF (°)	RPCF (°)	BPDF (°)	RPDF (°)
ex9°	9.22 ± 4.58^a^	4.60 ± 2.64^a^	3.35 ± 1.63^a^	1.05 ± 0.94^a^
	[4.5, 18.5]	[1.0, 10.5]	[1.0, 6.0]	[0.0, 4.0]
ex7°	11.80 ± 3.79^a^	5.20 ± 2.24^a^	3.55 ± 1.28^a^	1.65 ± 0.87
	[5.0, 18.0]	[2.0, 11.0]	[1.0, 6.0]	[0.0, 4.0]
ex5°	14.35 ± 4.27^a^	7.25 ± 2.38^a^	4.60 ± 1.60^a^	1.75 ± 1.21
	[6.0, 21.5]	[3.0, 11.0]	[1.5, 7.0]	[0.0, 4.5]
ex3°	17.10 ± 3.31	9.30 ± 4.70	5.20 ± 1.22	2.40 ± 0.99
	[12.5, 24.0]	[4.5, 23.0]	[3.5, 7.5]	[1.0, 4.5]
0°	19.05 ± 4.73	9.70 ± 4.27	5.90 ± 1.74	2.55 ± 1.73
	[12.0, 30.0]	[3.5, 20.0]	[3.0, 9.0]	[0.0, 6.0]
in3°	18.25 ± 4.36	9.55 ± 5.46	5.25 ± 1.24	2.10 ± 1.12
	[11.0, 27.5]	[5.5, 25.0]	[3.5, 8.0]	[1.5, 5.0]
in5°	15.20 ± 4.80^a^	7.80 ± 3.98^a^	4.80 ± 1.79^a^	1.92 ± 1.57
	[8.0, 25.0]	[2.0, 18.0]	[2.5, 8.5]	[0.0, 5.0]
in7°	13.25 ± 3.99^a^	6.15 ± 2.41^a^	2.95 ± 1.15^a^	1.50 ± 0.77
	[7.0, 20.5]	[2.0, 10.5]	[2.0, 6.0]	[0.0,,2.5]
in9°	11.00 ± 4.17^a^	6.50 ± 4.01^a^	2.55 ± 1.96^a^	0.25 ± 0.68^a^
	[4.0, 20.0]	[0.0, 10.0]	[1.0, 6.0]	[0.0, 2.0]
F	8.965	13.929	5.737	3.358
P	0.000	0.000	0.000	0.001

ex, extorsion; in, intorsion.

^a^P < 0.05 comparison with the value in the baseline (0°) by Tukey multiple comparison tests.

#### Foveal fusion

The average values and ranges of BPCF, RPCF, BPDF and RPDF are shown in Table 3. There were significant differences in BPCF, RPCF, BPDF and RPDF at different torsional angles (ANOVA test, F = 4.400, 3.748, 5.123, 2.003; P = 0.000, 0.000, 0.000, 0.049, respectively). In addition, the Tukey multiple comparison test showed that the average values of BPCF at extorsion and intorsion angles of 5°, 7°, and 9° decreased significantly compared to baseline (Fig. 3i–l). The average values of RPCF at extorsion 5°, extorsion and intorsion 5°, 7°, and 9° decreased significantly. Besides, the average values of BPDF and RPDF at extorsion and intorsion 7° and 9° decreased significantly compared to baseline (P < 0.05).

**Table 3:** BPCF, RPCF, BPDF and RPDF of f-F at different rotations.

Torsion	Convergent fusion (CF) n = 25		Divergent fusion (DF) n = 25	
	BPCF (°)	RPCF (°)	BPDF (°)	RPDF (°)
ex9°	10.55 ± 4.83^a^	5.40 ± 2.89^a^	3.80 ± 1.47^a^	1.40 ± 0.99^a^
	[4.0, 20.0]	[1.0, 11.0]	[2.0, 7.0]	[0.0, 3.0]
ex7°	11.45 ± 4.32^a^	6.05 ± 2.67^a^	3.55 ± 1.05^a^	1.27 ± 0.75^a^
	[7.0, 20.0]	[3.0, 15.5]	[2.5, 7.5]	[0.0, 4.0]
ex5°	12.65 ± 4.86^a^	6.80 ± 4.57^a^	4.10 ± 1.65	1.85 ± 1.22
	[6.0, 22.0]	[2.0, 18.0]	[2.0. 8.0]	[0.0, 3.5]
ex3°	15.60 ± 1.96	9.05 ± 2.44	4.95 ± 1.10	1.95 ± 0.82
	[10.0, 22.0]	[5.0, 15.0]	[2.0, 6.0]	[0.0, 3.5]
0°	16.70 ± 4.24	9.95 ± 4.06	5.30 ± 1.92	2.40 ± 1.27
	[10.0, 23.0]	[5.0, 18.5]	[2.5, 8.0]	[0.0, 4.0]
in3°	15.95 ± 3.36	9.55 ± 3.14	4.80 ± 0.80	1.75 ± 0.85
	[9.0, 22.0]	[4.0, 15.5]	[2.0, 6.5]	[0.0, 3.5]
in5°	14.00 ± 4.73^a^	7.40 ± 4.36	4.70 ± 2.08	1.85 ± 1.22
	[7.0, 21.5]	[1.0, 17.0]	[2.0, 6.0]	[0.0, 3.0]
in7°	13.45 ± 4.54^a^	6.53 ± 3.49^a^	3.52 ± 1.18^a^	1.20 ± 1.09^a^
	[5.0, 20.0]	[2.0, 11.5]	[2.0, 6.5]	[0.0, 2.0]
in9°	10.95 ± 4.60^a^	6.05 ± 3.97^a^	2.85 ± 0.93^a^	0.82 ± 0.78^a^
	[4.5, 20.0]	[2.0, 11.0]	[2.0, 6.0]	[0.0, 2.0]
F	4.400	3.748	5.123	2.003
P	0.000	0.000	0.000	0.049

ex, extorsion; in, intorsion.

^a^P < 0.05 comparison with the value in the baseline (0°) by Tukey’s multiple comparison tests.

Overall, there were no significant differences between fusion at extorsion and intorsion among fusion slides of different sizes.

#### The fusions of different visual fields

**Figure 4:**
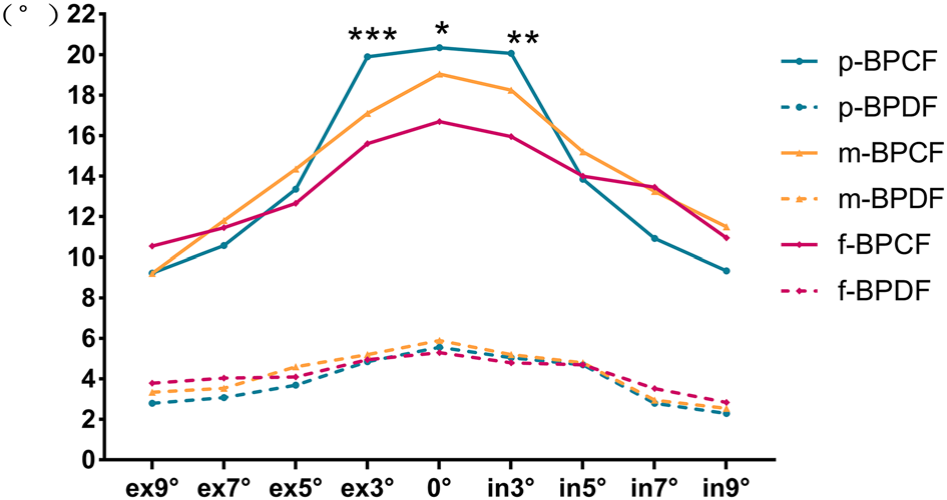
Break points of vergence fusion of the peripherial, macular and foveal fields at the different torsional angles. (ANOVA test, *P < 0.05, **P < 0.01, ***P < 0.001).

**Figure 5:**
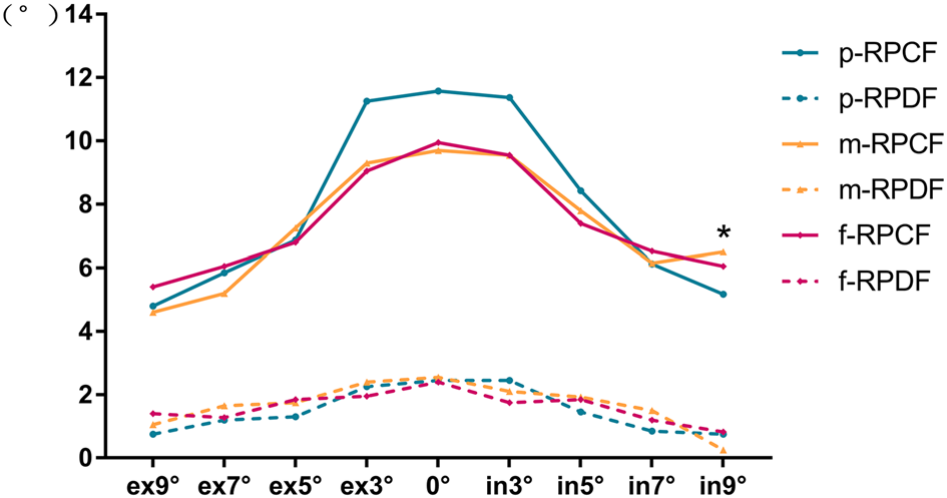
Recovery points of vergence fusion of the peripherial, macular and foveal fields at the different torsional angles.

The mean BP and RP of p-F, m-F, and f-F at different degrees of torsion are shown in Figs. 4 and 5. There were only significant differences in BPCF at 0°, intorsion 3° and extorsion 3° (ANOVA test, F = 3.981, 5.925, 9.850; P = 0.023, 0.004, 0.000, respectively) among p-F, m-F and f-F. Furthermore, the Tukey multiple comparison test showed significant differences between p-F and f-F (P = 0.037, 0.006, 0.000, 0.017, respectively).

### The effect of torsion on stereopsis

**Figure 6:**
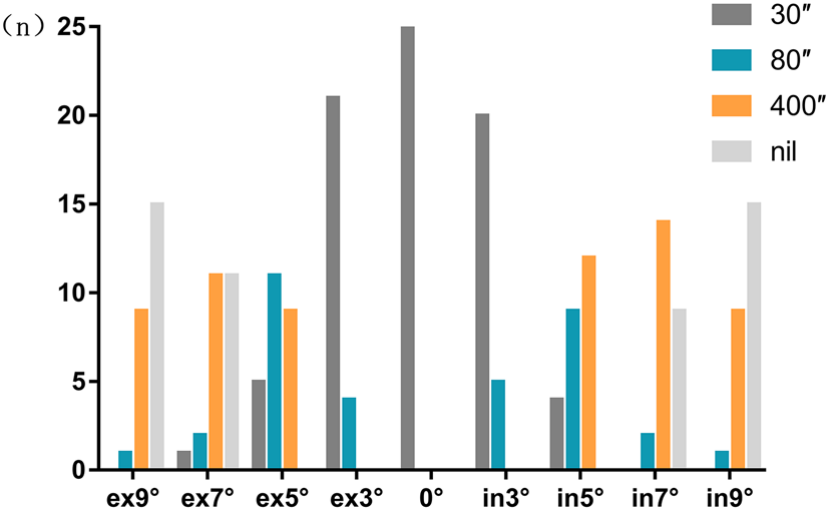
The proportionof the subjects having different scales of RDS at the different torsion.

All subjects had stereopsis 30″ tested by the synoptophore RDS slides at 0° torsion. The number of subjects having different RDS at different torsions is shown in Fig. 6. There was a significant difference in the proportion of subjects with different scales of RDS among different torsions (Fisher's exact test, P = 0.000).

The raw measurements for the 25 individuals were shown in the supplementary Table 1.

## Discussion

In our present study, the synoptophore assessment yielded mean break points for incyclofusion and excyclofusion of 16.35° and 15.85° in healthy subjects, consistent with the previous literature. We used three different sizes of fusion slides, 1° for foveal fusion, 5° for macular fusion and 11° for peripheral fusion. Although 11° fusion slides are not enough for the real peripheral visual field, they are still widely used to evaluate coarse fusion in clinical practice, while 1° fusion slides are for fine fusion. The break point represents the loss of fusion when the limit of fusional vergence is reached. The recovery point is determined by the person's ability to voluntarily converge/diverge in response to spatial disparity, representing the patient's ability to regain fusion voluntarily.

Georgievski et al. found that the binocular fusion in normal adults at 2° and 4° torsion was similar to that without torsion, but binocular fusion and stereopsis significantly reduced torsion beyond 6° torsion^8^. The break points and recovery points of convergent fusion in peripheral, macular and foveal visual fields decreased significantly at torsion ≥ 5º in our study, consistent with the literature. However, we found that the torsional effects on the divergent fusion of different visual fields were variable. The break points of divergent fusions decreased remarkably at torsion ≥ 3º, ≥ 5º and ≥ 7º for the peripheral, macular and foveal fields, respectively. The recovery points of divergent fusion were reduced at torsion ≥ 5º for the peripheral field, torsion ≥ 9° for the macular field and torsion ≥ 7º for the foveal field. We considered that torsion affected horizontal motor fusion even within the normal amplitude of cyclofusion. Peripheral fusion was wider than central fusion^12^, but its sensitivity to the rotation was as same as the central fusion. On the contrary, the divergent fusion of the peripheral field was more vulnerable than the foveal field. A comparison of the fusions at the same torsion among three visual fields revealed significant differences only for the break point of convergent fusion at baseline, intorsion 3° and extorsion 3°. This phenomenon may be attributed to: (1) the break point of peripheral fusion being more significantly reduced at torsion ≥ 5°, compared with macular and foveal fusion. (2) the actual peripheral areas of the retina are not completely stimulated by synoptophore slides. (3) the normal amplitudes of divergent fusion in three sizes of the stimulated area being narrow in healthy subjects.

It has been reported that after surgical treatment for acquired torsional diplopia, binocular vision could be improved from the peripheral fusion to the macular fusion^13^. Yagasaki et al.^14^ found that only the coarse stereopsis could be detected at a torsion angle of 10°, and fine stereopsis was completely lost at torsion beyond 4°. Although in our study RDS 30″ was detected in all subjects at baseline, almost half of all subjects at 5° torsion and all subjects at 9° torsion lost fine stereoacuity, while 60% of subjects at 9° torsion lost coarse stereoacuity.

Cyclodeviation is objectively measured by the fundus photographs during clinical practice. However, it should be borne in mind that by observing the angle between the fovea and optic disk, the patient's angle and the normal angle might overlap. Indeed, subjective torsion measurement is also important. Torsional diplopia might be absent in patients with cyclo- and vertical deviation due to the large range of cyclofusion or anomalous retinal correspondence^15^. Horizontal and vertical strabismus can be corrected with the prism, but cyclodeviation can only be resolved or improved by strabismic surgery. Based on our results, we suggest that for restoring and regaining fine binocular vision, a torsion ≥ 5° should be considered during strabismus surgery, and care should be taken to avoid increasing torsion.

In summary, the evaluation of ocular rotation and the appropriate surgical correction for torsion is conducive to rebuilding the peripheral and central fusion function and recovering stereovision. Limitations and shortcomings of the present study should be acknowledged, including the relatively small sample size and the lack of parameters assessing the effect of age on fusion function, which will be explored in our future studies.

## Supplementary Information

Supplementary Table 1.
