# Supplementary material for: The effects of torsion on horizontal motor fusion and stereopsis
Source: Sci Rep. 2023 Jan 16;13:792. doi: 10.1038/s41598-023-28169-z (PMC9842707; doi:10.1038/s41598-023-28169-z)
Supplement: Supplementary file 1 — Supplementary Table 1. [file 41598_2023_28169_MOESM1_ESM.docx]

supplementary table1. The raw measurements for the 25 individuals

| No. | age | sex | [p-BPCF@BASELINE](mailto:p-BPCF@BASELINE) | [p-RPCF@BASELINE](mailto:p-RPCF@BASELINE) | [p-BPDF@BASELINE](mailto:p-BPDF@BASELINE) | [p-RPDF@BASELINE](mailto:p-RPDF@BASELINE) | [p-BPCF IN3](mailto:p-BPCF@BASELINE) | [p-RPCF IN3](mailto:p-RPCF@BASELINE) | [p-BPDF IN3](mailto:p-BPDF@BASELINE) | [p-RPDF IN3](mailto:p-RPDF@BASELINE) | [p-BPCF EX3](mailto:p-BPCF@BASELINE) | [p-RPCF EX3](mailto:p-RPCF@BASELINE) | [p-BPDF EX3](mailto:p-BPDF@BASELINE) | [p-RPDF EX3](mailto:p-RPDF@BASELINE) |
| --- | --- | --- | --- | --- | --- | --- | --- | --- | --- | --- | --- | --- | --- | --- |
| 1 | 33 | 0 | 17 | 10 | 6 | 4 | 16.5 | 9 | 6 | 3.5 | 16 | 10 | 5 | 3 |
| 2 | 36 | 0 | 20 | 6 | 6 | 3 | 20 | 7 | 5 | 3 | 19 | 8 | 6 | 4 |
| 3 | 33 | 0 | 23 | 20 | 7.5 | 3 | 22 | 19.5 | 7 | 3.5 | 23 | 21 | 8 | 4 |
| 4 | 30 | 0 | 16 | 10 | 5 | 2 | 16 | 10 | 5 | 3 | 15 | 11 | 6 | 1 |
| 5 | 38 | 1 | 21 | 7 | 6 | 3 | 22.5 | 6 | 4.5 | 3 | 20 | 7 | 5 | 2 |
| 6 | 37 | 0 | 18 | 15 | 6 | 2 | 18 | 14 | 5 | 3 | 19 | 15 | 4 | 2.5 |
| 7 | 35 | 0 | 28 | 20 | 5 | 2 | 26 | 19.5 | 5 | 2 | 24 | 18 | 5 | 2 |
| 8 | 39 | 0 | 18 | 10 | 5 | 2 | 19 | 11 | 4.5 | 2.5 | 18 | 11 | 5 | 3 |
| 9 | 40 | 1 | 25 | 20 | 6 | 4 | 24.5 | 19 | 6 | 3 | 26 | 20 | 4.5 | 1 |
| 10 | 37 | 0 | 16 | 13 | 8 | 1.75 | 16.3 | 12 | 6 | 2 | 17 | 13 | 7 | 2.5 |
| 11 | 33 | 0 | 19 | 10 | 5 | 2 | 19 | 9 | 5 | 1 | 19 | 10 | 4 | 1 |
| 12 | 38 | 0 | 20 | 8 | 6 | 3 | 21 | 6 | 5 | 3 | 20 | 7 | 5 | 4 |
| 13 | 37 | 0 | 28 | 18 | 7.5 | 2 | 28.5 | 19 | 7 | 3 | 28 | 18 | 5 | 2 |
| 14 | 36 | 1 | 16 | 7 | 3 | 2 | 16 | 8 | 3 | 2 | 17 | 8 | 3 | 1 |
| 15 | 39 | 0 | 20 | 10 | 5 | 2 | 20 | 9 | 4 | 2 | 21 | 10 | 4 | 2 |
| 16 | 32 | 0 | 27 | 14 | 4 | 2.5 | 25 | 13 | 4 | 2 | 25 | 12 | 4 | 2 |
| 17 | 34 | 0 | 26 | 10 | 8 | 3 | 24 | 11 | 8 | 3.5 | 23 | 10 | 4 | 2 |
| 18 | 30 | 1 | 15.5 | 7 | 5 | 2 | 16 | 8 | 4 | 2 | 17 | 9 | 5.5 | 2 |
| 19 | 33 | 1 | 12 | 7.5 | 4 | 2 | 13 | 6.5 | 3 | 2 | 12.5 | 8.5 | 3 | 1.5 |
| 20 | 35 | 1 | 20 | 9 | 3.5 | 1.5 | 18 | 9 | 4 | 1 | 17 | 8 | 3 | 1 |
| 21 | 30 | 1 | 28 | 20 | 8 | 3 | 28.5 | 10.5 | 8 | 3 | 28 | 14 | 8 | 4 |
| 22 | 33 | 1 | 14 | 6 | 3 | 2 | 11 | 8.5 | 3 | 2 | 15 | 11 | 3 | 1 |
| 23 | 31 | 0 | 21.5 | 7 | 6 | 3.5 | 18 | 11.5 | 4 | 1 | 18 | 8 | 5 | 3 |
| 24 | 31 | 1 | 19 | 12 | 6 | 1.5 | 19 | 12 | 5.5 | 3 | 20 | 7 | 5 | 3 |
| 25 | 31 | 0 | 21 | 11 | 5 | 2.5 | 24 | 16 | 5 | 2 | 20 | 7 | 4.5 | 2 |

| No. | age | sex | [p-BPCF IN5](mailto:p-BPCF@BASELINE) | [p-RPCF IN5](mailto:p-RPCF@BASELINE) | [p-BPDF IN5](mailto:p-BPDF@BASELINE) | [p-RPDF IN5](mailto:p-RPDF@BASELINE) | [p-BPCF EX5](mailto:p-BPCF@BASELINE) | [p-RPCF EX5](mailto:p-RPCF@BASELINE) | [p-BPDF EX5](mailto:p-BPDF@BASELINE) | [p-RPDF EX5](mailto:p-RPDF@BASELINE) | [p-BPCF IN7](mailto:p-BPCF@BASELINE) | [p-RPCF IN7](mailto:p-RPCF@BASELINE) | [p-BPDF IN7](mailto:p-BPDF@BASELINE) | [p-RPDF IN7](mailto:p-RPDF@BASELINE) |
| --- | --- | --- | --- | --- | --- | --- | --- | --- | --- | --- | --- | --- | --- | --- |
| 1 | 33 | 0 | 11 | 8 | 5 | 2 | 12 | 7 | 5 | 3 | 8 | 5 | 4 | 2 |
| 2 | 36 | 0 | 11 | 6 | 6 | 3 | 8 | 3 | 4 | 2 | 8 | 5 | 3 | 1 |
| 3 | 33 | 0 | 20 | 14 | 7 | 2 | 19 | 9 | 7 | 1 | 15 | 7 | 3 | 1 |
| 4 | 30 | 0 | 10 | 4 | 4 | 2 | 6 | 2 | 2 | 0 | 8 | 3.5 | 2 | 0 |
| 5 | 38 | 1 | 10 | 5 | 4 | 1 | 8 | 4 | 5 | 1 | 10 | 4 | 3 | 1 |
| 6 | 37 | 0 | 14 | 10 | 4 | 0 | 12 | 10 | 1.5 | 0 | 12 | 9 | 1 | 1 |
| 7 | 35 | 0 | 22 | 7 | 5 | 1 | 22 | 8 | 4 | 1 | 16 | 8 | 3 | 0 |
| 8 | 39 | 0 | 13 | 10 | 4 | 2 | 17 | 9 | 5 | 2 | 10 | 7 | 4 | 1.5 |
| 9 | 40 | 1 | 22 | 18 | 6 | 3 | 21 | 17 | 6 | 3 | 18 | 14 | 5 | 2 |
| 10 | 37 | 0 | 15 | 12 | 7 | 2 | 15 | 11 | 4 | 2 | 15 | 10 | 4 | 1.5 |
| 11 | 33 | 0 | 14 | 8 | 2 | 0 | 16 | 8 | 2 | 0 | 14 | 7 | 2 | 0 |
| 12 | 38 | 0 | 15 | 4 | 5 | 2 | 16 | 4 | 2 | 0 | 13 | 3 | 2 | 0 |
| 13 | 37 | 0 | 22 | 17 | 7.5 | 2 | 20 | 10 | 8 | 2 | 15 | 9 | 6 | 0 |
| 14 | 36 | 1 | 8 | 6 | 3 | 1 | 6 | 2 | 2 | 0 | 7 | 2 | 1 | 0 |
| 15 | 39 | 0 | 18 | 10 | 5 | 0 | 12 | 8 | 1.5 | 0 | 15 | 7 | 1 | 0 |
| 16 | 32 | 0 | 12 | 7 | 4 | 0 | 20 | 10 | 4 | 1 | 9 | 9 | 3 | 0 |
| 17 | 34 | 0 | 7 | 4 | 8 | 3 | 7 | 2 | 3 | 3 | 5 | 2 | 4 | 2 |
| 18 | 30 | 1 | 11 | 7 | 4 | 2 | 12 | 5 | 3 | 2 | 8 | 4 | 2 | 2 |
| 19 | 33 | 1 | 10 | 5 | 2 | 1 | 9 | 4.5 | 2 | 2 | 6 | 4 | 1 | 1 |
| 20 | 35 | 1 | 12 | 6.5 | 2 | 0 | 9 | 4 | 3 | 1 | 6 | 3 | 2 | 1.5 |
| 21 | 30 | 1 | 22 | 18 | 7.5 | 3 | 22 | 17.5 | 8 | 3 | 18 | 14 | 6.5 | 2 |
| 22 | 33 | 1 | 7 | 4 | 2 | 0 | 6.5 | 3 | 1.5 | 0 | 5.5 | 2 | 1 | 0 |
| 23 | 31 | 0 | 13 | 7 | 3.5 | 2 | 11.5 | 5.5 | 3 | 1 | 12.5 | 11 | 2 | 0 |
| 24 | 31 | 1 | 13 | 7.5 | 4 | 1 | 14 | 3.5 | 2 | 1.5 | 10.5 | 7 | 2 | 1.5 |
| 25 | 31 | 0 | 14.5 | 7.5 | 6 | 1.5 | 13 | 5 | 4 | 1 | 8.5 | 6 | 2.5 | 0 |

| No. | age | sex | [p-BPCF EX7](mailto:p-BPCF@BASELINE) | [p-RPCF EX7](mailto:p-RPCF@BASELINE) | [p-BPDF EX7](mailto:p-BPDF@BASELINE) | [p-RPDF EX7](mailto:p-RPDF@BASELINE) | [p-BPCF IN9](mailto:p-BPCF@BASELINE) | [p-RPCF IN9](mailto:p-RPCF@BASELINE) | [p-BPDF IN9](mailto:p-BPDF@BASELINE) | [p-RPDF IN9](mailto:p-RPDF@BASELINE) | [p-BPCF EX9](mailto:p-BPCF@BASELINE) | [p-RPCF EX9](mailto:p-RPCF@BASELINE) | [p-BPDF EX9](mailto:p-BPDF@BASELINE) | [p-RPDF EX9](mailto:p-RPDF@BASELINE) |
| --- | --- | --- | --- | --- | --- | --- | --- | --- | --- | --- | --- | --- | --- | --- |
| 1 | 33 | 0 | 6 | 3 | 5 | 2 | 5 | 1 | 2 | 1 | 8 | 6 | 5 | 3 |
| 2 | 36 | 0 | 8 | 4 | 3.5 | 0 | 8 | 1 | 2 | 1 | 6 | 4 | 4 | 2 |
| 3 | 33 | 0 | 15 | 5 | 5 | 0 | 14 | 7 | 3 | 2 | 15 | 11 | 3 | 1 |
| 4 | 30 | 0 | 9 | 4 | 3 | 1 | 4 | 4 | 2 | 0 | 1 | 1 | 0 | 0 |
| 5 | 38 | 1 | 10 | 3 | 4 | 1 | 9 | 4 | 4 | 1 | 6 | 3 | 4 | 0 |
| 6 | 37 | 0 | 11 | 9 | 1 | 1 | 12 | 8 | 1 | 1 | 11 | 9 | 2 | 0 |
| 7 | 35 | 0 | 17 | 7 | 3 | 0 | 16 | 6 | 2 | 0 | 18 | 4 | 4 | 0 |
| 8 | 39 | 0 | 8 | 6.5 | 4 | 1 | 5 | 3 | 4 | 1 | 12 | 7 | 4 | 2 |
| 9 | 40 | 1 | 19.5 | 15 | 5 | 2 | 20 | 11 | 3 | 3 | 18.5 | 4 | 5 | 1 |
| 10 | 37 | 0 | 13 | 11 | 3 | 1 | 15 | 12 | 3 | 3 | 15 | 10 | 4 | 2 |
| 11 | 33 | 0 | 11 | 6 | 2 | 0 | 10 | 4 | 0 | 0 | 8 | 0 | 1 | 0 |
| 12 | 38 | 0 | 11 | 4 | 2 | 1 | 10 | 4 | 1 | 0 | 8 | 1 | 0 | 0 |
| 13 | 37 | 0 | 16 | 8 | 5 | 2 | 14 | 12 | 4 | 0 | 18 | 12 | 3 | 0 |
| 14 | 36 | 1 | 12 | 3 | 2 | 0 | 6 | 1 | 1 | 0 | 4 | 0 | 1 | 0 |
| 15 | 39 | 0 | 12 | 7 | 2 | 1 | 7 | 8 | 2 | 0 | 5 | 9 | 1 | 0 |
| 16 | 32 | 0 | 8 | 8 | 3 | 1 | 11 | 9 | 2 | 0 | 12 | 8 | 2 | 0 |
| 17 | 34 | 0 | 6 | 2 | 3 | 1 | 7 | 2 | 4 | 1 | 4 | 2 | 6 | 2.5 |
| 18 | 30 | 1 | 7 | 3 | 2 | 2 | 5 | 2 | 2 | 1 | 6 | 2 | 2 | 1 |
| 19 | 33 | 1 | 7 | 4 | 2 | 2 | 5 | 2 | 2 | 0 | 6 | 1 | 3 | 1 |
| 20 | 35 | 1 | 5 | 4 | 2 | 1 | 4 | 2.5 | 2 | 0 | 3 | 2 | 2 | 0 |
| 21 | 30 | 1 | 19.5 | 15.5 | 5 | 2 | 20 | 12 | 4 | 3 | 18.5 | 12 | 6 | 3 |
| 22 | 33 | 1 | 5 | 2 | 1 | 2 | 6 | 1 | 0 | 0 | 1 | 0 | 0 | 0 |
| 23 | 31 | 0 | 11 | 5.5 | 4 | 2 | 7.5 | 5 | 2 | 0 | 12 | 7 | 5 | 0.5 |
| 24 | 31 | 1 | 8.5 | 3 | 3.5 | 2 | 7 | 3.5 | 2.5 | 1 | 8.5 | 4 | 3 | 0 |
| 25 | 31 | 0 | 9 | 4 | 2 | 2 | 6.5 | 4 | 3 | 0 | 6 | 1 | 0 | 0 |

| No. | age | sex | [m-BPCF@BASELINE](mailto:m-BPCF@BASELINE) | [m-RPCF@BASELINE](mailto:m-RPCF@BASELINE) | [m-BPDF@BASELINE](mailto:m-BPDF@BASELINE) | [m-RPDF@BASELINE](mailto:m-RPDF@BASELINE) | [m-BPCF IN3](mailto:p-BPCF@BASELINE) | [m-RPCF IN3](mailto:p-RPCF@BASELINE) | [m-BPDF IN3](mailto:p-BPDF@BASELINE) | [m-RPDF IN3](mailto:p-RPDF@BASELINE) | [m-BPCF EX3](mailto:p-BPCF@BASELINE) | [m-RPCF EX3](mailto:p-RPCF@BASELINE) | [m-BPDF EX3](mailto:p-BPDF@BASELINE) | [m-RPDF EX3](mailto:p-RPDF@BASELINE) |
| --- | --- | --- | --- | --- | --- | --- | --- | --- | --- | --- | --- | --- | --- | --- |
| 1 | 33 | 0 | 15 | 8 | 8 | 3 | 15 | 8 | 8 | 2 | 15 | 8 | 7 | 3 |
| 2 | 36 | 0 | 24 | 10 | 8 | 4 | 22 | 10 | 6 | 2 | 24 | 23 | 7.5 | 2 |
| 3 | 33 | 0 | 23 | 19 | 6 | 4 | 23 | 15 | 5 | 2 | 22 | 11 | 5.5 | 2 |
| 4 | 30 | 0 | 18 | 8 | 5 | 1 | 17 | 8 | 5 | 2 | 15 | 8 | 5 | 3 |
| 5 | 38 | 1 | 14 | 6 | 3 | 1 | 11 | 5.5 | 3.5 | 1.5 | 12.5 | 7 | 7 | 2 |
| 6 | 37 | 0 | 16 | 4 | 8 | 3 | 17 | 5.5 | 5 | 2 | 15 | 7 | 6 | 4.5 |
| 7 | 35 | 0 | 30 | 12 | 5 | 1 | 25 | 8 | 7 | 1.5 | 20 | 13 | 5 | 4.5 |
| 8 | 39 | 0 | 14 | 10 | 6 | 5 | 13 | 9 | 4 | 2 | 17 | 5 | 3.5 | 2 |
| 9 | 40 | 1 | 21 | 14 | 6 | 4 | 20 | 12 | 7.5 | 2.5 | 18 | 9 | 4 | 1 |
| 10 | 37 | 0 | 15 | 12 | 4 | 1 | 14 | 10 | 5 | 2 | 13 | 9 | 7 | 3 |
| 11 | 33 | 0 | 19 | 9 | 6 | 1 | 20 | 8.5 | 6 | 1.5 | 16 | 11 | 5 | 2 |
| 12 | 38 | 0 | 18 | 6 | 6 | 2 | 16 | 10 | 5 | 2 | 15 | 14 | 5.5 | 3 |
| 13 | 37 | 0 | 27 | 20 | 8 | 0 | 27 | 9 | 6 | 1.5 | 21 | 13 | 5 | 4.5 |
| 14 | 36 | 1 | 18 | 9 | 4 | 1 | 19 | 9 | 4 | 1.5 | 16 | 5 | 4 | 2 |
| 15 | 39 | 0 | 18 | 10 | 3 | 1 | 16 | 12 | 3.5 | 1.5 | 14 | 4.5 | 3.5 | 1 |
| 16 | 32 | 0 | 24 | 10 | 6 | 2 | 22 | 15 | 6.5 | 2 | 21 | 11 | 5 | 3 |
| 17 | 34 | 0 | 12 | 3.5 | 9 | 6 | 14 | 8 | 5 | 5 | 15 | 9 | 6 | 2 |
| 18 | 30 | 1 | 22 | 7 | 7 | 5 | 21 | 11 | 6 | 3 | 22 | 13 | 4 | 1 |
| 19 | 33 | 1 | 18 | 8 | 6 | 4 | 16 | 9 | 4 | 2 | 15 | 13 | 3.5 | 3 |
| 20 | 35 | 1 | 15 | 8 | 4 | 2 | 13 | 8 | 4 | 1.5 | 15 | 6 | 3.5 | 1 |
| 21 | 30 | 1 | 30 | 20 | 9 | 7 | 27.5 | 25 | 6.5 | 5 | 24 | 8 | 7 | 4.5 |
| 22 | 33 | 1 | 12 | 3.5 | 3 | 0 | 11 | 5.5 | 5 | 1.5 | 12.5 | 4.5 | 3.5 | 1 |
| 23 | 31 | 0 | 13.5 | 6.5 | 5 | 2 | 17 | 6 | 6 | 1.5 | 15 | 6 | 5 | 2 |
| 24 | 31 | 1 | 20 | 9 | 6 | 3 | 20 | 6 | 4 | 2 | 17 | 6.5 | 5 | 2 |
| 25 | 31 | 0 | 20 | 10 | 6.5 | 1 | 20 | 6 | 3.5 | 1.5 | 17.5 | 8 | 7 | 2.5 |

| No. | age | sex | [m-BPCF IN5](mailto:p-BPCF@BASELINE) | [m-RPCF IN5](mailto:p-RPCF@BASELINE) | [m-BPDF IN5](mailto:p-BPDF@BASELINE) | [m-RPDF IN5](mailto:p-RPDF@BASELINE) | [m-BPCF EX5](mailto:p-BPCF@BASELINE) | [m-RPCF EX5](mailto:p-RPCF@BASELINE) | [m-BPDF EX5](mailto:p-BPDF@BASELINE) | [m-RPDF EX5](mailto:p-RPDF@BASELINE) | [m-BPCF IN7](mailto:p-BPCF@BASELINE) | [m-RPCF IN7](mailto:p-RPCF@BASELINE) | [m-BPDF IN7](mailto:p-BPDF@BASELINE) | [m-RPDF IN7](mailto:p-RPDF@BASELINE) |
| --- | --- | --- | --- | --- | --- | --- | --- | --- | --- | --- | --- | --- | --- | --- |
| 1 | 33 | 0 | 10 | 6 | 5 | 2 | 13 | 8 | 5 | 2 | 13 | 4 | 4 | 1 |
| 2 | 36 | 0 | 18 | 9 | 6 | 5 | 20 | 3 | 6 | 3 | 18 | 3 | 6 | 2 |
| 3 | 33 | 0 | 22 | 18 | 5 | 1 | 21 | 11 | 5 | 1 | 16 | 10.5 | 5 | 3 |
| 4 | 30 | 0 | 16 | 8 | 3 | 1 | 16 | 10 | 3 | 1 | 9 | 9 | 3 | 1 |
| 5 | 38 | 1 | 8 | 3 | 3 | 1 | 7 | 6 | 3 | 1 | 9 | 5 | 2 | 2 |
| 6 | 37 | 0 | 12 | 6 | 4 | 2 | 11 | 6 | 6 | 2 | 11 | 5 | 5 | 2 |
| 7 | 35 | 0 | 25 | 8 | 4 | 5 | 20 | 10 | 4 | 0 | 20.5 | 9 | 3 | 0 |
| 8 | 39 | 0 | 9 | 8 | 5 | 3 | 12 | 10 | 5 | 2 | 10 | 9 | 2 | 1 |
| 9 | 40 | 1 | 20 | 14 | 5 | 3 | 18 | 10 | 5 | 4 | 19 | 9 | 2 | 2.5 |
| 10 | 37 | 0 | 14 | 6 | 4 | 2 | 13 | 7 | 4 | 2 | 15 | 8 | 3 | 2 |
| 11 | 33 | 0 | 16 | 10 | 4 | 0 | 16 | 8 | 4 | 0 | 14 | 8 | 3 | 2 |
| 12 | 38 | 0 | 16 | 5 | 6 | 2 | 13 | 5 | 6 | 2 | 15 | 6 | 4 | 3 |
| 13 | 37 | 0 | 21 | 15 | 8 | 2 | 20 | 10 | 7 | 3 | 20 | 8 | 3 | 2 |
| 14 | 36 | 1 | 14 | 8 | 2.5 | 1 | 6 | 3 | 2 | 1 | 12 | 2 | 3 | 0 |
| 15 | 39 | 0 | 16 | 8 | 2.5 | 0 | 11 | 6 | 1.5 | 0 | 9 | 3 | 2 | 0 |
| 16 | 32 | 0 | 20 | 6 | 4 | 1 | 18 | 8 | 4 | 1 | 14 | 4 | 2 | 1 |
| 17 | 34 | 0 | 8 | 2 | 8 | 4 | 11 | 7 | 4 | 1 | 7 | 5 | 3 | 0.5 |
| 18 | 30 | 1 | 15 | 7 | 7 | 1 | 12 | 6 | 5 | 2 | 8 | 5 | 3.5 | 2 |
| 19 | 33 | 1 | 11 | 5 | 5 | 2 | 14 | 5 | 6 | 3 | 12 | 5 | 2 | 2 |
| 20 | 35 | 1 | 13 | 4 | 5 | 1.5 | 15 | 6 | 6 | 2 | 14 | 6 | 2 | 1 |
| 21 | 30 | 1 | 25 | 18 | 8.5 | 5 | 21.5 | 11 | 7 | 4.5 | 20.5 | 10 | 3 | 3 |
| 22 | 33 | 1 | 8 | 2 | 2.5 | 0 | 6 | 4 | 1.5 | 0 | 7 | 2 | 2 | 0 |
| 23 | 31 | 0 | 10 | 4.5 | 4 | 0 | 16 | 8 | 6 | 2 | 12 | 5 | 2.5 | 2 |
| 24 | 31 | 1 | 15 | 7 | 4 | 2 | 15.5 | 7 | 5 | 1 | 12.5 | 6 | 2 | 1 |
| 25 | 31 | 0 | 18 | 7.5 | 5 | 1.5 | 12.5 | 6.5 | 4 | 3 | 14 | 7 | 2 | 1.5 |

| No. | age | sex | [m-BPCF EX7](mailto:p-BPCF@BASELINE) | [m-RPCF EX7](mailto:p-RPCF@BASELINE) | [m-BPDF EX7](mailto:p-BPDF@BASELINE) | [m-RPDF EX7](mailto:p-RPDF@BASELINE) | [m-BPCF IN9](mailto:p-BPCF@BASELINE) | [m-RPCF IN9](mailto:p-RPCF@BASELINE) | [m-BPDF IN9](mailto:p-BPDF@BASELINE) | [m-RPDF IN9](mailto:p-RPDF@BASELINE) | [m-BPCF EX9](mailto:p-BPCF@BASELINE) | [m-RPCF EX9](mailto:p-RPCF@BASELINE) | [m-BPDF EX9](mailto:p-BPDF@BASELINE) | [m-RPDF EX9](mailto:p-RPDF@BASELINE) |
| --- | --- | --- | --- | --- | --- | --- | --- | --- | --- | --- | --- | --- | --- | --- |
| 1 | 33 | 0 | 11 | 7 | 5 | 2 | 13 | 10 | 6 | 2 | 7 | 3 | 3 | 1 |
| 2 | 36 | 0 | 8 | 2 | 6 | 2 | 18 | 10 | 3 | 0 | 8 | 1 | 6 | 2 |
| 3 | 33 | 0 | 16 | 8 | 6 | 2 | 18 | 8 | 3 | 0 | 15 | 9 | 5 | 2 |
| 4 | 30 | 0 | 10 | 5 | 4 | 2 | 10 | 3 | 4 | 0 | 9 | 4 | 3 | 1 |
| 5 | 38 | 1 | 11 | 5 | 3 | 1 | 10 | 5 | 4 | 0 | 10 | 5 | 3 | 0 |
| 6 | 37 | 0 | 11 | 5 | 3 | 2 | 12 | 4 | 2 | 0 | 12 | 6 | 5 | 2 |
| 7 | 35 | 0 | 18 | 3 | 2 | 1 | 20 | 8 | 3 | 0 | 18.5 | 3 | 2 | 0 |
| 8 | 39 | 0 | 10 | 4 | 3 | 2 | 5 | 3 | 2 | 0 | 9 | 6 | 4 | 2 |
| 9 | 40 | 1 | 12 | 6 | 2 | 1 | 18 | 13 | 2 | 0 | 13 | 8 | 2 | 0.5 |
| 10 | 37 | 0 | 13 | 7 | 3 | 2 | 14 | 5 | 5 | 1 | 12 | 6 | 4 | 2 |
| 11 | 33 | 0 | 17 | 11 | 2 | 0 | 12 | 8 | 2 | 0.5 | 10 | 10.5 | 2 | 0 |
| 12 | 38 | 0 | 10 | 3 | 3 | 0 | 13 | 10 | 1 | 1 | 9 | 1 | 4 | 0 |
| 13 | 37 | 0 | 18 | 5 | 5 | 2 | 20 | 10 | 3 | 0 | 16 | 7 | 6 | 2 |
| 14 | 36 | 1 | 9 | 2 | 3 | 2 | 12 | 6.5 | 2 | 1 | 5 | 2 | 2 | 1 |
| 15 | 39 | 0 | 9 | 3 | 2 | 1 | 10 | 8 | 1 | 0 | 8 | 4 | 1 | 0 |
| 16 | 32 | 0 | 18 | 8 | 3 | 2 | 12 | 8 | 2 | 1 | 15 | 6 | 2 | 1 |
| 17 | 34 | 0 | 5 | 4 | 5 | 4 | 6 | 4 | 2 | 0 | 5.5 | 2 | 6 | 3 |
| 18 | 30 | 1 | 7 | 5 | 4 | 2 | 5 | 4 | 4 | 0 | 5 | 4 | 4 | 0 |
| 19 | 33 | 1 | 11 | 6 | 3 | 1 | 10 | 9 | 3.5 | 0 | 10 | 3 | 2 | 1 |
| 20 | 35 | 1 | 12 | 5 | 4 | 2 | 7 | 8 | 2.5 | 0 | 9 | 3 | 2 | 0 |
| 21 | 30 | 1 | 18 | 11 | 6.5 | 4 | 5 | 4 | 1 | 0 | 6 | 10.5 | 6.5 | 3 |
| 22 | 33 | 1 | 5 | 2 | 1 | 0 | 5 | 0 | 1 | 0 | 4.5 | 1 | 1 | 0 |
| 23 | 31 | 0 | 12 | 3 | 1 | 0 | 10 | 8 | 1.5 | 0 | 4.5 | 3.5 | 4 | 1.5 |
| 24 | 31 | 1 | 13 | 4 | 3 | 2 | 6 | 4 | 1.5 | 0 | 5 | 3 | 2 | 0 |
| 25 | 31 | 0 | 11 | 6 | 5 | 2.5 | 4 | 2 | 2 | 0 | 4.5 | 3.5 | 2.5 | 1 |

| No. | age | sex | [f-BPCF@BASELINE](mailto:f-BPCF@BASELINE) | [f-RPCF@BASELINE](mailto:f-RPCF@BASELINE) | [f-BPDF@BASELINE](mailto:f-BPDF@BASELINE) | [f-RPDF@BASELINE](mailto:f-RPDF@BASELINE) | [f-BPCF IN3](mailto:p-BPCF@BASELINE) | [f-RPCF IN3](mailto:p-RPCF@BASELINE) | [f-BPDF IN3](mailto:p-BPDF@BASELINE) | [f-RPDF IN3](mailto:p-RPDF@BASELINE) | [f-BPCF EX3](mailto:p-BPCF@BASELINE) | [f-RPCF EX3](mailto:p-RPCF@BASELINE) | [f-BPDF EX3](mailto:p-BPDF@BASELINE) | [f-RPDF EX3](mailto:p-RPDF@BASELINE) |
| --- | --- | --- | --- | --- | --- | --- | --- | --- | --- | --- | --- | --- | --- | --- |
| 1 | 33 | 0 | 11 | 7 | 5 | 3 | 11 | 7 | 4 | 2 | 10 | 7 | 5 | 3 |
| 2 | 36 | 0 | 20 | 18.5 | 8 | 4 | 19 | 11 | 5 | 3.5 | 16 | 11 | 6 | 3.5 |
| 3 | 33 | 0 | 20 | 17 | 4 | 1 | 19 | 13 | 6 | 1 | 16 | 12 | 6 | 4 |
| 4 | 30 | 0 | 17 | 8 | 4 | 1 | 16 | 7 | 6 | 1 | 16 | 11 | 6 | 2 |
| 5 | 38 | 1 | 14 | 9 | 4 | 1 | 15 | 6 | 4 | 1 | 16 | 10 | 6 | 3.5 |
| 6 | 37 | 0 | 20 | 5 | 8 | 3 | 19 | 5 | 6 | 3 | 17 | 9 | 6 | 1 |
| 7 | 35 | 0 | 20 | 9 | 2.5 | 0 | 19 | 6 | 6.5 | 0 | 17 | 8 | 6 | 2 |
| 8 | 39 | 0 | 10 | 8 | 4 | 2 | 9 | 7 | 4 | 2 | 13 | 6 | 6 | 2 |
| 9 | 40 | 1 | 23 | 15 | 5 | 4 | 22 | 13 | 4 | 2 | 15 | 7 | 5 | 1 |
| 10 | 37 | 0 | 12 | 9 | 4 | 2 | 14 | 8 | 4 | 0.5 | 17 | 15 | 4 | 2 |
| 11 | 33 | 0 | 15 | 8 | 4 | 1 | 13 | 7 | 3 | 2 | 13 | 13 | 6 | 3 |
| 12 | 38 | 0 | 10 | 8 | 7 | 4 | 10 | 7 | 4 | 1.5 | 11 | 11 | 4 | 0 |
| 13 | 37 | 0 | 23 | 16 | 8 | 3 | 17 | 12 | 5 | 2 | 22 | 7 | 5 | 2 |
| 14 | 36 | 1 | 13 | 9 | 2.5 | 1 | 15 | 12 | 5.5 | 0 | 14 | 8 | 6 | 3 |
| 15 | 39 | 0 | 20 | 12 | 5 | 2 | 18 | 11 | 2 | 2 | 13 | 8 | 5 | 2 |
| 16 | 32 | 0 | 20 | 10 | 6 | 3 | 18 | 8 | 4 | 2 | 13 | 8 | 6 | 0 |
| 17 | 34 | 0 | 12 | 5 | 8 | 4 | 14 | 7 | 3 | 3 | 14 | 9 | 5 | 2 |
| 18 | 30 | 1 | 17 | 9 | 6 | 4 | 16 | 8 | 6 | 2 | 15 | 8 | 6 | 3.5 |
| 19 | 33 | 1 | 18 | 7 | 5 | 3 | 17 | 4 | 4 | 2 | 16 | 7 | 5 | 0 |
| 20 | 35 | 1 | 19 | 8 | 5.5 | 2 | 18 | 15.5 | 6 | 2 | 15 | 6 | 3.5 | 1 |
| 21 | 30 | 1 | 23 | 18.5 | 8 | 4 | 20 | 15.5 | 6.5 | 3.5 | 22 | 15 | 2 | 1 |
| 22 | 33 | 1 | 10 | 5 | 2.5 | 0 | 9 | 14 | 4 | 0 | 18 | 6 | 2 | 0 |
| 23 | 31 | 0 | 18 | 6.5 | 5 | 3 | 16 | 4.5 | 6.5 | 4 | 16 | 5 | 4 | 2 |
| 24 | 31 | 1 | 15.5 | 9.5 | 5 | 3 | 20 | 15.5 | 5 | 1 | 17 | 10 | 5 | 3 |
| 25 | 31 | 0 | 17 | 12 | 6.5 | 2 | 15 | 15 | 6 | 1 | 18 | 9.5 | 3 | 2 |

| No. | age | sex | [f-BPCF](mailto:p-BPCF@BASELINE)  [IN5](mailto:p-BPCF@BASELINE) | [f-RPCF IN5](mailto:p-RPCF@BASELINE) | [f-BPDF IN5](mailto:p-BPDF@BASELINE) | [f-RPDF](mailto:p-RPDF@BASELINE)  [IN5](mailto:p-RPDF@BASELINE) | [f-BPCF EX5](mailto:p-BPCF@BASELINE) | [f-RPCF EX5](mailto:p-RPCF@BASELINE) | [f-BPDF EX5](mailto:p-BPDF@BASELINE) | [f-RPDF EX5](mailto:p-RPDF@BASELINE) | [f-BPCF IN7](mailto:p-BPCF@BASELINE) | [f-RPCF IN7](mailto:p-RPCF@BASELINE) | [f-BPDF IN7](mailto:p-BPDF@BASELINE) | [f-RPDF IN7](mailto:p-RPDF@BASELINE) |
| --- | --- | --- | --- | --- | --- | --- | --- | --- | --- | --- | --- | --- | --- | --- |
| 1 | 33 | 0 | 11 | 7 | 4 | 3 | 11 | 5 | 5 | 2 | 9 | 8 | 4 | 2 |
| 2 | 36 | 0 | 10 | 1 | 6 | 4 | 12 | 8 | 3 | 2 | 8 | 3 | 2 | 2 |
| 3 | 33 | 0 | 20 | 16 | 4 | 1 | 20 | 18 | 3 | 2 | 20 | 11 | 4 | 1 |
| 4 | 30 | 0 | 15 | 8 | 4 | 1 | 16 | 6 | 4 | 2 | 11 | 4 | 2 | 1 |
| 5 | 38 | 1 | 7 | 2 | 2 | 0 | 15 | 8 | 6 | 3.5 | 15 | 10 | 3 | 1 |
| 6 | 37 | 0 | 10 | 8 | 6 | 2 | 8 | 6 | 4 | 2 | 16 | 2 | 2 | 0 |
| 7 | 35 | 0 | 20 | 6 | 5 | 0 | 8 | 6 | 5 | 0 | 18 | 8 | 2.5 | 0 |
| 8 | 39 | 0 | 8 | 7 | 4 | 2 | 16 | 6 | 3.5 | 2 | 7 | 6.5 | 4 | 2 |
| 9 | 40 | 1 | 22 | 16 | 5 | 3 | 22 | 15 | 4 | 3.5 | 20 | 11.5 | 4 | 2 |
| 10 | 37 | 0 | 14 | 7 | 3 | 1 | 17 | 8 | 3 | 1 | 15 | 6 | 3.5 | 1 |
| 11 | 33 | 0 | 14 | 9 | 3 | 1 | 10 | 6 | 2 | 0 | 11 | 4.5 | 4 | 0 |
| 12 | 38 | 0 | 12 | 8 | 2 | 2 | 8 | 5 | 4 | 2 | 11 | 5 | 2 | 1 |
| 13 | 37 | 0 | 21.5 | 17 | 6 | 2 | 8 | 4 | 6 | 3 | 15 | 9.5 | 6 | 2 |
| 14 | 36 | 1 | 10 | 4 | 6 | 1 | 16 | 10 | 2.5 | 0 | 9 | 5 | 3 | 0 |
| 15 | 39 | 0 | 12 | 4 | 6 | 0 | 10 | 4 | 6 | 1 | 13 | 5 | 3 | 1 |
| 16 | 32 | 0 | 18 | 6 | 4 | 2 | 18 | 10 | 5.5 | 2 | 19 | 6 | 4 | 1 |
| 17 | 34 | 0 | 8 | 4 | 6 | 3 | 6 | 5 | 8 | 3 | 14.5 | 7 | 6 | 2 |
| 18 | 30 | 1 | 16 | 5 | 5 | 3 | 7 | 2 | 2 | 3 | 16 | 5 | 4 | 3 |
| 19 | 33 | 1 | 16 | 6 | 6 | 2 | 10 | 6 | 6 | 3 | 13 | 4 | 3 | 3 |
| 20 | 35 | 1 | 15 | 7 | 7 | 3 | 8 | 6 | 2 | 1 | 12 | 5 | 3 | 2 |
| 21 | 30 | 1 | 21.5 | 17 | 6 | 4 | 22 | 4 | 6 | 2 | 20 | 11.5 | 6.5 | 3 |
| 22 | 33 | 1 | 7 | 1 | 6 | 0.5 | 10.5 | 6 | 3 | 0 | 7 | 2 | 2 | 0 |
| 23 | 31 | 0 | 14 | 7 | 3 | 1 | 12 | 8 | 4 | 2 | 5 | 8 | 2 | 0 |
| 24 | 31 | 1 | 13 | 4 | 4.5 | 2 | 8 | 4 | 2 | 2 | 15 | 10 | 6.5 | 0 |
| 25 | 31 | 0 | 15 | 8 | 4 | 3 | 18 | 4 | 3 | 2 | 17 | 5.5 | 2 | 0 |

| No. | age | sex | [f-BPCF EX7](mailto:p-BPCF@BASELINE) | [f-RPCF EX7](mailto:p-RPCF@BASELINE) | [f-BPDF EX7](mailto:p-BPDF@BASELINE) | [f-RPDF](mailto:p-RPDF@BASELINE)  [EX7](mailto:p-RPDF@BASELINE) | [f-BPCF IN9](mailto:p-BPCF@BASELINE) | [f-RPCF IN9](mailto:p-RPCF@BASELINE) | [f-BPDF IN9](mailto:p-BPDF@BASELINE) | [f-RPDF IN9](mailto:p-RPDF@BASELINE) | [f-BPCF EX9](mailto:p-BPCF@BASELINE) | [f-RPCF EX9](mailto:p-RPCF@BASELINE) | [f-BPDF EX9](mailto:p-BPDF@BASELINE) | [f-RPDF EX9](mailto:p-RPDF@BASELINE) |
| --- | --- | --- | --- | --- | --- | --- | --- | --- | --- | --- | --- | --- | --- | --- |
| 1 | 33 | 0 | 12 | 8 | 4 | 2 | 10 | 8 | 3 | 2 | 11 | 7 | 4 | 3 |
| 2 | 36 | 0 | 7 | 3 | 6 | 2 | 6 | 2 | 6 | 0 | 5 | 1 | 4 | 2 |
| 3 | 33 | 0 | 18 | 11 | 4 | 2 | 20 | 16 | 4 | 1 | 16 | 10 | 4 | 2 |
| 4 | 30 | 0 | 13 | 7 | 3 | 2 | 10 | 5 | 4 | 1 | 12 | 6 | 5 | 2.5 |
| 5 | 38 | 1 | 10 | 6 | 2.5 | 1 | 14 | 9 | 2 | 1 | 9 | 4 | 3 | 1 |
| 6 | 37 | 0 | 10 | 6 | 3 | 1 | 14 | 8 | 3 | 1 | 20 | 6 | 5 | 1 |
| 7 | 35 | 0 | 12 | 5 | 4 | 0 | 16 | 7 | 2 | 0 | 8 | 4 | 3 | 0 |
| 8 | 39 | 0 | 9 | 6 | 5 | 2 | 7 | 6 | 4 | 2 | 8 | 6 | 4 | 3 |
| 9 | 40 | 1 | 20 | 9 | 4 | 2 | 19 | 14 | 2 | 2 | 15 | 11 | 2 | 1 |
| 10 | 37 | 0 | 12.5 | 8 | 4 | 2 | 14 | 7 | 3 | 1 | 12 | 7 | 4 | 3 |
| 11 | 33 | 0 | 9 | 5 | 3 | 2 | 11 | 4 | 2 | 0 | 8 | 4 | 3 | 0 |
| 12 | 38 | 0 | 10 | 5 | 3 | 0 | 11 | 5 | 2 | 1 | 11 | 5 | 7 | 3 |
| 13 | 37 | 0 | 17 | 10 | 2.5 | 1 | 16 | 8.5 | 2 | 2 | 18 | 10 | 5 | 0 |
| 14 | 36 | 1 | 7 | 3 | 3 | 1 | 8 | 4 | 2 | 0 | 6 | 2 | 2 | 1 |
| 15 | 39 | 0 | 9 | 3 | 4 | 0.5 | 12 | 4 | 3 | 1 | 8 | 2 | 4 | 1 |
| 16 | 32 | 0 | 19 | 9 | 3 | 1 | 8 | 8 | 4 | 1 | 18 | 8 | 3 | 1 |
| 17 | 34 | 0 | 7 | 2 | 7.5 | 4 | 4.5 | 2 | 2 | 0 | 4 | 1 | 7 | 3 |
| 18 | 30 | 1 | 14 | 6 | 3 | 2 | 15 | 4 | 5 | 0 | 12 | 6 | 3 | 2 |
| 19 | 33 | 1 | 8 | 5 | 3 | 0 | 11.5 | 3 | 2 | 2 | 4 | 4 | 2 | 0 |
| 20 | 35 | 1 | 8 | 4 | 2.5 | 0.5 | 11 | 3.5 | 3 | 1 | 6 | 4 | 2 | 1 |
| 21 | 30 | 1 | 20 | 15.5 | 4 | 4 | 14 | 11 | 2 | 0 | 20 | 11 | 7 | 3 |
| 22 | 33 | 1 | 7 | 3 | 3 | 0 | 5.5 | 2 | 2 | 0 | 4 | 1 | 2 | 0 |
| 23 | 31 | 0 | 8 | 4 | 3 | 0 | 5 | 4 | 3.5 | 1 | 12 | 4 | 2 | 0.5 |
| 24 | 31 | 1 | 7 | 3 | 2.5 | 0 | 6 | 4 | 2 | 0.5 | 11.5 | 8 | 6 | 1 |
| 25 | 31 | 0 | 12.5 | 4.5 | 2.5 | 0 | 5 | 2 | 2 | 0 | 5 | 3 | 2 | 0 |

| No. | age | sex | Stereopsis@baseline | in3° Stereopsis | in5° Stereopsis | in7° Stereopsis | in9° Stereopsis | ex3° Stereopsis | ex5° Stereopsis | ex7° Stereopsis | ex9° Stereopsis |
| --- | --- | --- | --- | --- | --- | --- | --- | --- | --- | --- | --- |
| 1 | 33 | 0 | 30 | 30 | 400 | 0 | 0 | 30 | 400 | 400 | 0 |
| 2 | 36 | 0 | 30 | 30 | 400 | 0 | 0 | 30 | 400 | 0 | 0 |
| 3 | 33 | 0 | 30 | 30 | 30 | 400 | 400 | 30 | 30 | 30 | 80 |
| 4 | 30 | 0 | 30 | 30 | 400 | 0 | 0 | 80 | 400 | 0 | 0 |
| 5 | 38 | 1 | 30 | 30 | 80 | 400 | 400 | 30 | 400 | 400 | 400 |
| 6 | 37 | 0 | 30 | 30 | 400 | 0 | 0 | 30 | 400 | 0 | 0 |
| 7 | 35 | 0 | 30 | 80 | 400 | 0 | 0 | 30 | 400 | 0 | 0 |
| 8 | 39 | 0 | 30 | 30 | 400 | 0 | 0 | 30 | 400 | 400 | 0 |
| 9 | 40 | 1 | 30 | 30 | 30 | 400 | 0 | 30 | 30 | 400 | 0 |
| 10 | 37 | 0 | 30 | 30 | 400 | 0 | 0 | 30 | 400 | 400 | 0 |
| 11 | 33 | 0 | 30 | 30 | 400 | 400 | 400 | 30 | 400 | 400 | 400 |
| 12 | 38 | 0 | 30 | 80 | 80 | 400 | 0 | 30 | 80 | 80 | 400 |
| 13 | 37 | 0 | 30 | 30 | 80 | 0 | 0 | 30 | 80 | 400 | 0 |
| 14 | 36 | 1 | 30 | 30 | 400 | 400 | 0 | 30 | 80 | 80 | 400 |
| 15 | 39 | 0 | 30 | 30 | 30 | 400 | 400 | 80 | 30 | 400 | 400 |
| 16 | 32 | 0 | 30 | 30 | 80 | 400 | 400 | 30 | 80 | 400 | 400 |
| 17 | 34 | 0 | 30 | 30 | 80 | 80 | 80 | 30 | 80 | 0 | 400 |
| 18 | 30 | 1 | 30 | 80 | 400 | 400 | 0 | 80 | 80 | 400 | 400 |
| 19 | 33 | 1 | 30 | 30 | 400 | 400 | 400 | 30 | 80 | 0 | 0 |
| 20 | 35 | 1 | 30 | 30 | 400 | 400 | 400 | 30 | 80 | 0 | 400 |
| 21 | 30 | 1 | 30 | 80 | 30 | 80 | 0 | 30 | 30 | 400 | 0 |
| 22 | 33 | 1 | 30 | 30 | 80 | 400 | 400 | 30 | 80 | 0 | 0 |
| 23 | 31 | 0 | 30 | 30 | 80 | 400 | 0 | 80 | 80 | 0 | 0 |
| 24 | 31 | 1 | 30 | 80 | 80 | 400 | 400 | 30 | 30 | 0 | 0 |
| 25 | 31 | 0 | 30 | 30 | 80 | 0 | 0 | 30 | 80 | 0 | 0 |
